# *Wolbachia* in the spittlebug *Prosapia ignipectus*: Variable infection frequencies, but no apparent effect on host reproductive isolation

**DOI:** 10.1002/ece3.7782

**Published:** 2021-07-04

**Authors:** Timothy B. Wheeler, Vinton Thompson, William R. Conner, Brandon S. Cooper

**Affiliations:** ^1^ Division of Biological Sciences University of Montana Missoula MT USA; ^2^ Division of Invertebrate Zoology American Museum of Natural History New York NY USA

**Keywords:** Cercopidae, cytoplasmic incompatibility, endosymbiosis, host–microbe interaction, speciation

## Abstract

Animals serve as hosts for complex communities of microorganisms, including endosymbionts that live inside their cells. *Wolbachia* bacteria are perhaps the most common endosymbionts, manipulating host reproduction to propagate. Many *Wolbachia* cause cytoplasmic incompatibility (CI), which results in reduced egg hatch when uninfected females mate with infected males. *Wolbachia* that cause intense CI spread to high and relatively stable frequencies, while strains that cause weak or no CI tend to persist at intermediate, often variable, frequencies. *Wolbachia* could also contribute to host reproductive isolation (RI), although current support for such contributions is limited to a few systems. To test for *Wolbachia* frequency variation and effects on host RI, we sampled several local *Prosapia ignipectus* (Fitch) (Hemiptera: Cercopidae) spittlebug populations in the northeastern United States over two years, including closely juxtaposed Maine populations with different monomorphic color forms, “black” and “lined.” We discovered a group‐B *Wolbachia* (*w*Pig) infecting *P. ignipectus* that diverged from group‐A *Wolbachia*—like model *w*Mel and *w*Ri strains in *Drosophila*—6 to 46 MYA. Populations of the sister species *Prosapia bicincta* (Say) from Hawaii and Florida are uninfected, suggesting that *P. ignipectus* acquired *w*Pig after their initial divergence. *w*Pig frequencies were generally high and variable among sites and between years. While phenotyping *w*Pig effects on host reproduction is not currently feasible, the *w*Pig genome contains three divergent sets of CI loci, consistent with high *w*Pig frequencies. Finally, Maine monomorphic black and monomorphic lined populations of *P. ignipectus* share both *w*Pig and mtDNA haplotypes, implying no apparent effect of *w*Pig on the maintenance of this morphological contact zone. We hypothesize *P. ignipectus* acquired *w*Pig horizontally as observed for many *Drosophila* species, and that significant CI and variable transmission produce high but variable *w*Pig frequencies.

## Graphical abstract

We report that a group‐B *Wolbachia* (*w*Pig) infects the spittlebug *Prosapia ignipectus*. *w*Pig was likely recently acquired and has little apparent effect on the maintenance of a *P. ignipectus* morphological contact zone in Maine, USA. The *w*Pig genome contains three sets of loci associated with cytoplasmic incompatibility (CI), and we hypothesize that significant CI and variable transmission produce high but variable *w*Pig frequencies in nature.

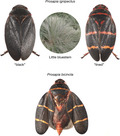

## 1 INTRODUCTION

Animals interact with microorganisms that influence their behavior, physiology, and fitness (Brownlie et al., 2009; Fredericksen et al., 2017; Gould et al., 2018; Hague, Caldwell, et al., 2020; Hurst & Jiggins, 2000; McFall‐Ngai et al., 2013). These include associations between hosts and vertically transmitted endosymbionts that live inside their cells (McCutcheon et al., 2019). Hosts may acquire endosymbionts cladogenically from common ancestors (Koga et al., 2013; Raychoudhury et al., 2009; Toju et al., 2013), from sister species via hybridization and introgression (Cooper et al., 2019), or horizontally in ways that are not fully understood (Ahmed et al., 2015; Huigens et al., 2000; O’Neill et al., 1992; Turelli et al., 2018). While few examples exist, endosymbionts can contribute to host reproductive isolation (RI) (Coyne & Orr, 2004; Matute & Cooper, 2021), highlighting the importance of discovering and characterizing endosymbiont–host associations.

Maternally transmitted *Wolbachia* bacteria are widely distributed (Weinert et al., 2015; Werren et al., 2008; Zug & Hammerstein, 2012), infecting many arthropods and two groups of parasitic nematodes (Bandi et al., 1998), making *Wolbachia* the most common known endosymbionts in nature. In *Drosophila*, introgressive and horizontal *Wolbachia* acquisition seem to predominate (Conner et al., 2017; Cooper et al., 2019; Turelli et al., 2018), but cladogenic acquisition during host speciation has been observed in other taxa (Gerth & Bleidorn, 2017; Raychoudhury et al., 2009). Many *Wolbachia* manipulate host reproduction to propagate in host populations. For example, many strains cause cytoplasmic incompatibility (CI) that reduces the egg hatch of uninfected embryos fertilized by *Wolbachia*‐infected sperm (Hoffmann & Turelli, 1997). However, if females are also infected, the embryos survive, “rescuing” CI and promoting *Wolbachia* spread to high frequencies (Barton & Turelli, 2011; Hoffmann et al., 1990; Kriesner et al., 2013; Turelli & Hoffmann, 1995).

*Wolbachia* may contribute to host RI (Coyne & Orr, 2004; Matute & Cooper, 2021), with the best evidence coming from *Drosophila*. *Wolbachia* contribute to assortative mating and postzygotic isolation between co‐occurring *D. paulistorum* semispecies (Miller et al., 2010), and to reinforcement of isolation between uninfected *D. subquinaria* and *Wolbachia*‐infected *D. recens* (Jaenike et al., 2006; Shoemaker et al., 1999). In contrast, *Wolbachia* do not contribute to RI in the *D. yakuba* clade, which includes *w*Yak‐infected *D. yakuba*, *w*San‐infected *D. santomea*, and *w*Tei‐infected *D. teissieri* (Cooper et al., 2017). Thus, while some results from *Drosophila* strongly support contributions of *Wolbachia* to RI, and interest in the possibility of such effects remains high, it is unknown whether *Wolbachia* effects on RI are common in nature.

*Wolbachia* frequencies differ significantly among infected host taxa, ranging from very low to obligately fixed infections (Bandi et al., 1998; Cooper et al., 2017; Kriesner et al., 2013; Miller et al., 2010). *Wolbachia* effects on reproduction (e.g., CI) and fitness (e.g., fecundity effects, Weeks et al., 2007), in combination with imperfect maternal transmission, govern its frequencies in host populations (Caspari & Watson, 1959; Hoffmann et al., 1990). Intensive sampling of a few systems has revealed both stable and variable *Wolbachia* frequencies within host populations. *Wolbachia* that cause intense CI, like *w*Ri in *Drosophila simulans*, persist at high and relatively stable frequencies, balanced by imperfect maternal transmission (Kriesner et al., 2013; Turelli et al., 2018). In contrast, *Wolbachia* that cause weak or no CI tend to occur at variable intermediate frequencies (Cooper et al., 2017; Hamm et al., 2014; Hoffmann et al., 1996; Kriesner et al., 2016; Meany et al., 2019) via effects on host fitness that are mostly unknown (Brownlie et al., 2009; Hague et al., 2021; Teixeira et al., 2008; Weeks et al., 2007). These strains include *w*Mel‐like *Wolbachia* with frequencies that vary spatially in *D. melanogaster* and *D. yakuba* (Hague, Caldwell, et al., 2020; Kriesner et al., 2016), and temporally in *D. yakuba* and *D. santomea* (Cooper et al., 2017; Hague, Mavengere, et al., 2020). In all but a few other systems, limited sampling has left a gap in knowledge about whether *Wolbachia* frequency variation is common (Cattel et al., 2016; Hamm et al., 2014; Hughes, Allsopp, et al., 2011; Hughes, Ren, et al., 2011; Ross et al., 2020; Schuler et al., 2016).

**FIGURE 1:**
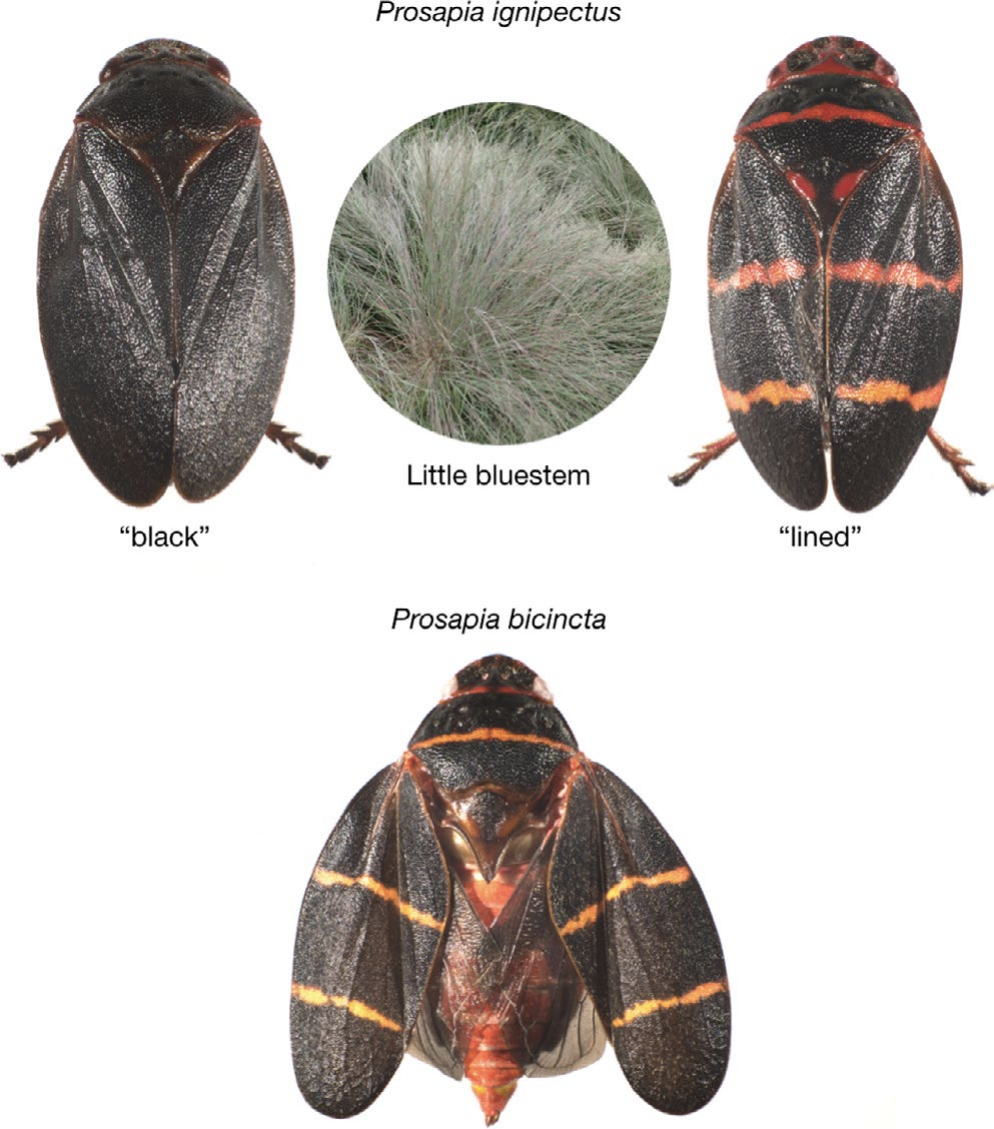
Sister species *Prosapia ignipectus* and *Prosapia* *bicincta* have conspicuous dorsal coloration. All *P. bicincta* individuals have a single narrow transverse orange line across the widest part of the pronotum and a pair of narrow orange lines across the elytra. Most *P. ignipectus* individuals have a solid black dorsal surface, but in Maine some *P. ignipectus* have *P. bicincta*‐like coloration. *P. ignipectus* is monophagous on the late season C4 perennial grass *Schizachyrium scoparium* (Little bluestem). Little bluestem photo by Krzysztof Ziarnek, Kenraiz (CC BY‐SA 4.0, https://creativecommons.org/licenses/by‐sa/4.0)

*Prosapia ignipectus* (Fitch) (Hemiptera: Cercopidae) is one of about 14 species of *Prosapia* and one of two commonly found in the United States, the other being its sister species *P. bicincta* (Say) (Hamilton, 1977). *P. ignipectus* occurs in southern Ontario, Canada, and the northeastern United States from Minnesota to Maine (Carvalho & Webb, 2005; Hamilton, 1977, 1982; Peck, 1999; Thompson & Carvalho, 2016). These species vary in male genital morphology and in associations with host plants, with *P. ignipectus* monophagous on the late season C4 perennial grass *Schizachyrium scoparium* (Little bluestem) (Hamilton, 1982; Thompson, 2004) and *P. bicincta* polyphagous on a variety of C4 grasses, but not including Little bluestem (Fagan & Kuitert, 1969; Thompson, 2004). Both species have conspicuous dorsal coloration, standing out against their respective host plants. All *P. bicincta* individuals have a single narrow transverse orange line across the widest part of the pronotum and a pair of narrow orange lines across the elytra. Most *P. ignipectus* individuals have a solid black dorsal surface, but in Maine some *P. ignipectus* have *P. bicincta*‐like coloration (Figure 1). Notably, only 10 km separate monomorphic black and monomorphic lined *P. ignipectus* populations in western Maine, with little evidence of a hybrid zone and no obvious physical barriers to mixing across the boundary (Thompson & Carvalho, 2016). This morphological contact zone has persisted for at least 90 years. About 45 km southwest of this abrupt transition between aposematic color forms, three other *P. ignipectus* populations were found to be polymorphic with both black and lined forms—these populations are surrounded by monomorphic black populations. It has been hypothesized that *Wolbachia* may contribute to host RI and to preservation of the sharp Maine morphological contact zone (Thompson & Carvalho, 2016).

Here, we use collections of *P. ignipectus* from several sites in the northeastern United States across two years, in combination with collections of *P. bicincta* from Hawaii and Florida, United States, to assess modes of *Wolbachia* acquisition and to test for *Wolbachia* frequency variation through space and time. By sampling monomorphic black and lined populations and typing both *Wolbachia* and mtDNA haplotypes, we also test for contributions of *Wolbachia* to the *P. ignipectus* morphological contact zone. Finally, we generate whole genome *Wolbachia* data for phylogenetic analysis and to search for loci associated with inducing and rescuing CI (Beckmann et al., 2017; LePage et al., 2017; Shropshire et al., 2018). While we cannot currently test *P. ignipectus* for CI in the laboratory, CI‐causing *Wolbachia* are predicted to occur at high infection frequencies and to have specific loci associated with CI in their genomes.

## 2 METHODS

### 2.1 Sampling

We netted specimens from Little bluestem; sorted them by species, sex, and color form; and preserved them in 95% ethanol. The 2019 specimens (*N* = 4 sites) were collected on August 23. The 2020 specimens (*N* = 9 sites) were collected on August 9 (Silver Lake, NH), August 17 (Wonalancet, NH), and August 20 (all Maine localities) (Table S1). Collection sites were on the verges of public rights of way or privately owned land. In two cases (New Vineyard and New Portland), they correspond to sites reported in Thompson and Carvalho (2016). Specimens were collected near the height of abundance for *P. ignipectus*, which starts to emerge in adult form in late July and early August. We also sampled three additional spittlebug species at these sites: *Lepyronia quadrangularis* (Say) (*N* = 25), *Philaenus spumarius* (L.) (*N* = 5), and *Philaenarcys killa* (Hamilton) (*N* = 24), all of the family Aphrophoridae. Like, *P. ignipectus*, *P. killa* a is monophage on Little bluestem. *L. quadrangularis* is a polyphage but often abundant on Little bluestem. *P. spumarius* is an extreme polyphage, with a preference for forbs (herbaceous perennial dicots) but is occasionally collected from Little bluestem in the company of *P. ignipectus*. By screening them for *Wolbachia*, we tested for the possibility of horizontal *Wolbachia* transfer through plant interactions (Chrostek et al., 2017). Lastly, because identification of infections in sister hosts enables formal analysis of modes of *Wolbachia* acquisition (Conner et al., 2017; Cooper et al., 2019; Raychoudhury et al., 2009; Turelli et al., 2018), we also obtained samples of the sister species *P. bicincta* from Hawaii (*N* = 60) and Florida (*N* = 40) to screen for infections. *P. bicincta* is native to the southeastern United States (Fagan & Kuitert, 1969; Thompson & Carvalho, 2016), but has recently been introduced into the Kona Region of Big Island, Hawaii (Thorne et al., 2018).

### 2.2 *Wolbachia* typing

We generated whole‐genome *Wolbachia* data to type the *Wolbachia* infecting *P. ignipectus* and to search for loci associated with CI. We extracted 800ng of high molecular weight DNA (Qiagen Genomic‐tip 20/G; Qiagen, Germany) from one black New Vineyard female (see below), and then input and sequenced it (Ligation Sequencing Kit, SQK‐LSK109; FLO‐MIN106 flow cell) for 48 hr (Oxford Nanopore Technologies). We mapped raw nanopore reads (5.8Gb of data) to all known *Wolbachia* sequences (NCBI taxid 953) with BLASTn and extracted reads where at least 60% of their length mapped (qcovs ≥ 60). We then corrected and assembled reads using canu 2.1.1 (Koren et al., 2017, 2018; Nurk et al., 2020) and polished the *Wolbachia* assembly using nanopolish 0.13.2 (Loman et al., 2015). We annotated our *Wolbachia* assembly plus the genomes of model group‐A (*w*Mel, Wu et al., 2004; and *w*Ri, Klasson et al., 2009) and group‐B (*w*Pip‐Pel, Klasson et al., 2008; and *w*Mau, Meany et al., 2019) strains using Prokka v.1.11 (Seemann, 2014). We used only genes present in single copy and with identical lengths in all genomes. To assess the quality of our assembly, we excluded *w*Pig and repeated this with only *w*Mel, *w*Ri, *w*Pip, and *w*Mau.

Preliminary analysis of a few loci placed the *P. ignipectus Wolbachia* in group‐B (see below), but we performed Bayesian analyses using the GTR + Γ + I model for sequence evolution using whole‐genome data to confirm this (Höhna et al., 2016). Genes were concatenated and partitioned by codon position, with a rate multiplier, σ, assigned to each partition to accommodate variable substitution rates. We used flat, symmetrical Dirichlet priors on the stationary base frequencies, π, and the relative rate parameters, η, of the GTR model (i.e., Dirichlet(1,1,1…)). As in Turelli et al. (2018), we used a Γ(2,1) hyperprior on the shape parameter, α, of the discrete‐Γ model (adopting the conventional assumption that the β rate parameter equals α, so that the mean rate is 1 (Yang, 1994). The Γ model for rate variation assigns significant probability near zero when the α < 1 (accommodating invariant sites). The Γ(2,1) hyperprior on α assigns 95% probability to the interval (0.36, 4.74), allowing for small and large values. Four independent runs for each gene set produced concordant topologies. We diagnosed MCMC performance using Tracer 1.7 (Rambaut et al., 2014).

### 2.3 *Wolbachia* and mtDNA haplotyping of black and lined color morphs

To confirm that the same *Wolbachia* strain infects different *P. ignipectus* populations and color morphs, we amplified and Sanger sequenced five protein‐coding *Wolbachia* genes (*coxA*, *hcpA*, *fbpA*, *ftsZ*, and *wsp*) in both directions (Eurofins Genomics LLC, Louisville, Kentucky)(see below, Table S2). We also amplified and Sanger sequenced *gatb*, but sequence quality was consistently too low to include in our analyses. Samples included one infected female of each color form (black or lined), from each of the four populations (Carthage, New Portland, New Vineyard, and Strong) sampled in both years (Table S1).

To specifically assess whether *Wolbachia* might contribute to the morphological contact zone between New Vineyard (monomorphic black) and New Portland (monomorphic lined) *P. ignipectus*, we amplified and Sanger sequenced the *cytochrome C oxidase I* (*CoI*) mitochondrial locus from one male and one female from these populations, with the exception of one (New Vineyard black male) that did not produce a usable sequence. We also produced *CoI* sequences for one black and one lined female from the polymorphic Strong population. Covariance of *Wolbachia* and mtDNA haplotypes with *P. ignipectus* color forms would support a potential role for *Wolbachia* in maintaining the morphological contact zone.

We visually inspected each sequence for quality and ambiguities, and consensus sequences were used as queries for a BLASTn search and the NCBI “nr” database to confirm that orthologous genes were amplified (Altschul et al., 1990). We then used the “multiple locus query” function of the multi locus sequence typing (MLST) database to type *Wolbachia* (Baldo et al., 2006). Together, these data enable us to test for differentiation in *Wolbachia* and mtDNA between populations and color forms, including between populations monomorphic for different color forms separated by only 10 km in Maine.

### 2.4 Analysis of CI loci

Recent work has identified CI‐causing factors (*cifs*) associated with WO prophage in *Wolbachia* genomes (Beckmann et al., 2017; LePage et al., 2017; Shropshire & Bordenstein, 2019; Shropshire et al., 2020; Shropshire et al., 2018). Two genes (*cifA/B*) transgenically expressed in male *D. melanogaster* induce CI, while one gene (*cifA*) expressed in females rescues it. To identify *cif* loci, we used BLASTn to search for *cif* homologs in our whole‐genome raw reads, querying the Type 1 *cif* pair in *w*Mel, the Type 2 pair in *w*Ri, the Type 3 pair in *w*No, the Type 4 pair in *w*Pip, and the Type 5 pair in *w*Stri (Bing et al., 2020; Lindsey et al., 2018; Martinez et al., 2020). We later broadened our search for Type 1 pairs by querying *w*Pip and *w*NPa pairs (Gerth & Bleidorn, 2017; Klasson et al., 2008). For each Type, we extracted raw reads that covered at least 40% of the genes. We then corrected and assembled the reads with canu 2.1.1 (Koren et al., 2017, 2018; Nurk et al., 2020), producing sequences with about a 1% error rate. We limit our analyses to the discovery of *cif* types, since we did not generate additional sequence data to further correct the long reads. The assembled genes were compared to those in Martinez et al. (2020).

### 2.5 Analysis of *Wolbachia* frequency variation

To test for *Wolbachia* frequency variation, we extracted DNA from many individuals from each collection using a standard squish buffer protocol and identified *Wolbachia* infections using polymerase chain reaction (PCR) (Simpliamp ThermoCycler; Applied Biosystems, Singapore) (Meany et al., 2019). We amplified the *Wolbachia* surface protein (*wsp*) (Braig et al., 1998) and arthropod‐specific 28S rDNA, which served as a positive control (Baldo et al., 2006) (Table S2). PCR products were visualized using 1% agarose gels. Assuming a binomial distribution, we estimated exact 95% confidence intervals for *Wolbachia* frequencies for each collection. We used Fisher's exact test (FET) to determine differences in frequencies among sites, between years, between sexes, and between color forms.

## 3 RESULTS

### 3.1 *Prosapia* *ignipectus* likely acquired its group‐B *Wolbachia* following initial divergence from *P. bicincta*

**FIGURE 2:**
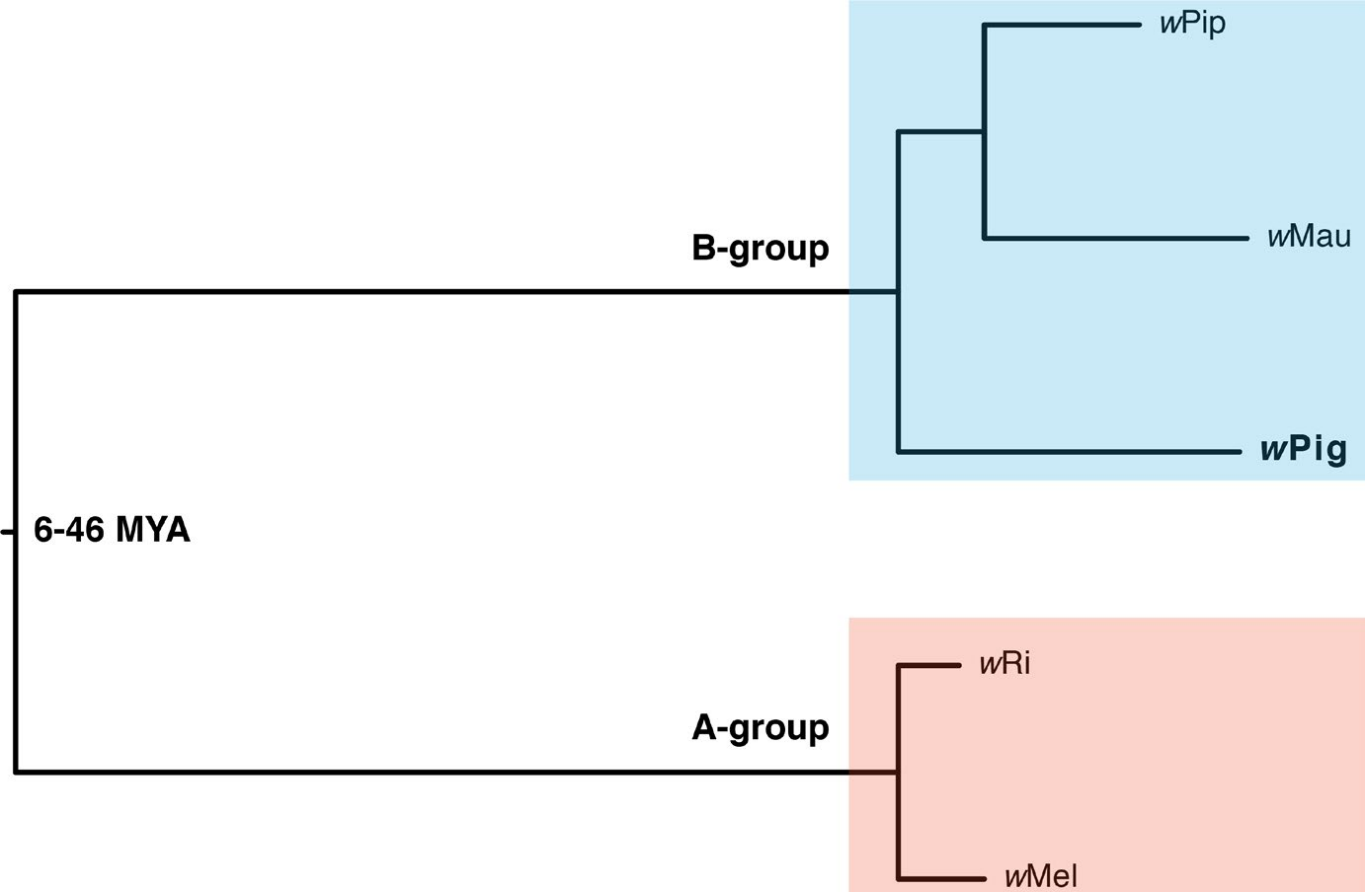
An estimated phylogram for model group‐A (*w*Ri, Klasson et al., 2009; *w*Mel, Wu et al., 2004) and group‐B (*w*Pip_Pel, Klasson et al., 2008; *w*Mau, Meany et al., 2019) *Wolbachia*, plus *w*Pig. All nodes have Bayesian posterior probabilities of 1. The divergence time of groups A and B is superimposed from (Meany et al., 2019). The phylogram shows significant variation in the substitution rates across branches, with long branches separating groups A and B

Across all samples, *Wolbachia* infection frequency (*p*) in *P. ignipectus* is high (*p* = 0.93 [0.90, 0.95]; *N* = 486). Based on five Sanger sequenced loci, the multiple sequence query of the MLST database supports that a group‐B strain, most closely related to *Wolbachia* in Chloropidae (Diptera) (ID 93, ST 104), infects our *P. ignipectus* samples—we call this strain *w*Pig. Preliminary phylogenetic analyses using only our five Sanger sequenced genes also placed *w*Pig in group B. Our draft *w*Pig assembly size (1.32Mb, N50 = 91,011) falls in the range of complete *Wolbachia* genomes (e.g., *w*Mel at 1.26 Mb and *w*Ri at 1.44 Mb), despite its fragmentation (50 contigs). In total, we extracted 65 single‐copy homologs of equal length (43,473 total bp) for our phylogenetic analysis, which also places *w*Pig in group B (Figure 2). When excluding the *w*Pig genome, we were able to extract an additional 135 homologs (167,241 bp) from *w*Mel, *w*Ri, *w*Pip, and *w*Mau. This indicates that significant residual error in the *w*Pig assembly reduces the number of homologs meeting our equal length criteria for inclusion. Finer placement of *w*Pig among group‐B strains will require the generation of short‐read data to further correct our draft *w*Pig assembly. Thus, we do not attempt to place *w*Pig precisely among group‐B strains.

None of the *P. bicincta* samples from Hawaii and Florida were *Wolbachia* infected. Even if some *P. bicincta* are *Wolbachia* infected, as previously reported for one individual used as a PCR control in another study (Anderson et al., 2019), *Wolbachia* infection frequency (*p*) must be very low across the *P. bicincta* range, given our species estimate and credible interval (*p* = 0.0 [0.0, 0.04]; *N* = 100), keeping in mind the possibility that the Hawaiian population may have experienced a recent bottleneck during introduction and may not be representative of the species in the native range. Very low frequency *Wolbachia* infections in global *P. bicincta* populations, in combination with generally high *w*Pig frequencies in *P. ignipectus*, indicate that *P. ignipectus* likely acquired *w*Pig after its initial divergence from *P. bicincta*, although it is also possible that *P. bicincta* lost its resident *Wolbachia* following cladogenic acquisition. Because testing predictions about modes of *Wolbachia* acquisition requires formal analysis of *Wolbachia*, host nuclear, and host mtDNA phylograms and chronograms, we are unable to distinguish between introgressive and horizontal *w*Pig transfer (Conner et al., 2017; Cooper et al., 2019; Gerth & Bleidorn, 2017; Raychoudhury et al., 2009; Turelli et al., 2018). We discuss this further below.

Of the additional species we netted from Little bluestem, all *L. quadrangularis* were uninfected (*p* = 0.0 [0.0, 0.14]; *N* = 25), all *P*. *spumarius* were infected (*p* = 1.0 [0.48, 1.0]; *N* = 5), and only one *P. killa* individual was infected (*p* = 0.04 [0.001, 0.21]; *N* = 24). Because *Wolbachia* that infect *P. spumarius* and *w*Pig in *P. ignipectus* are both at high frequency, we also typed the *Wolbachia* infecting *P*. *spumarius* to determine whether a *w*Pig‐like variant infects this host species. The multiple sequence query in the MLST database supports that a different group‐B strain, most closely related to the thrip species *Aptinothrips rufus* (ID 1945, ST 509) infects *P. spumarius*. Generating more sequence data will be required to resolve the phylogenetic relationships of these and other group‐B strains, including *Wolbachia* in *P. spumarius* (Lis et al., 2015).

### 3.2 No apparent effect of *w*Pig on the maintenance of the morphological *P. ignipectus* contact zone

**FIGURE 3:**
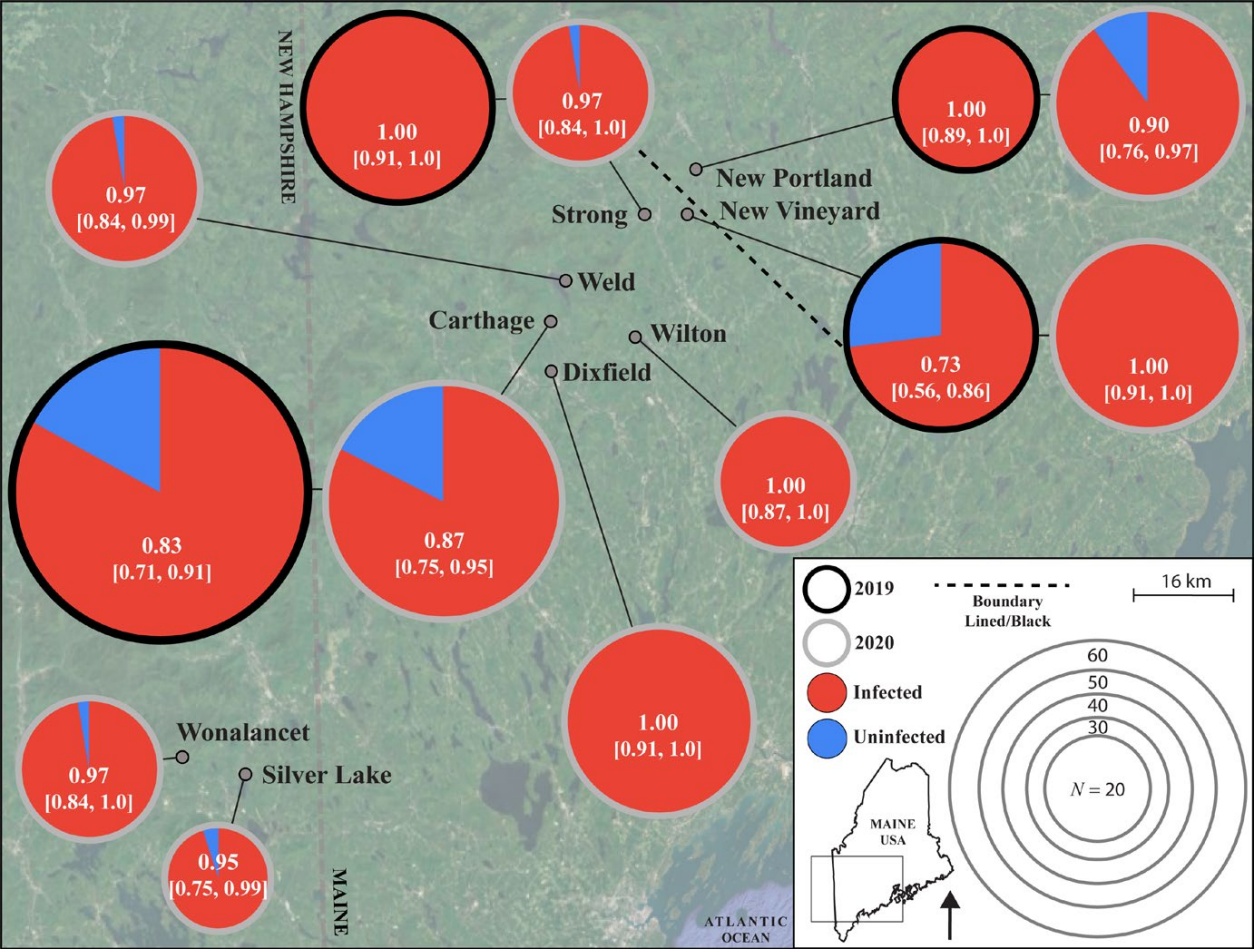
*w*Pig frequency varies through space and time. Circle size denotes sample size, with outline and fill color denoting sampling year and infection status, respectively. Sample means and 95% binomial confidence intervals are reported for each sample. The dashed back line denotes the geographical separation of monomorphic black and monomorphic lined *Prosapia ignipectus* populations

The Strong, Carthage, and Dixfield *P. ignipectus* populations (Figure 3) were polymorphic for the black and lined forms (Figure 1, Table S1), like three populations close to Rumford, Maine sampled in earlier work (Thompson & Carvalho, 2016). This set of mixed color form populations runs roughly from Rumford northeast to Strong, but not to the sharp boundary dividing the monomorphic black New Vineyard population from the monomorphic lined New Portland population. It has the appearance of a hybrid zone, but one that does not reach the definitive boundary between the forms. The existence of distinct color forms both within and between the populations sampled facilitated investigation of the relationship, if any, between *Wolbachia* infection and patterns of color form occurrence.

We found no evidence for *w*Pig genetic differentiation between *P. ignipectus* populations or color forms. Regions of the five *w*Pig genes we sequenced were identical, except for a single nucleotide position in *wsp*, where the Strong lined sample differed from all others. In addition to populations sharing *w*Pig type based on MLST loci, *w*Pig frequency did not vary between color forms (black: *p* = 0.93 [0.90, 0.95], *N* = 338; lined: *p* = 0.92 [0.86, 0.96], *N* = 123; FET, *P* = 0.69), between color forms for males only (black: *p* = 0.84 [0.75, 0.90], *N* = 98; lined: *p* = 0.90 [0.79, 0.97], *N* = 51; FET, *P* = 0.33), or among females (black: *p* = 0.97 [0.94, 0.99], *N* = 240; lined: *p* = 0.93 [0.85, 0.98], *N* = 72; FET, *P* = 0.19), across all samples. *w*Pig frequency also did not differ between New Vineyard (monomorphic black) and New Portland (monomorphic lined) populations (FET, *P* = 0.16).

We found no evidence for differentiation in *CoI* mtDNA haplotype between the New Vineyard and New Portland *P. ignipectus* populations, where all samples were identical across the 680 bp that we recovered. The black and lined females from the polymorphic Strong population also did not differ from each other, or from other populations, across this region. Thus, *w*Pig and mtDNA haplotypes were not differentiated between populations or color forms.

Our mtDNA haplotypes are also very similar to ten *P. ignipectus* samples included in the Barcode of Life Database (BOLD) (Foottit et al., 2014). A single base‐pair insertion present in all of our samples is absent from all ten BOLD samples. Four other sites in *CoI* that are polymorphic among the BOLD samples are fixed in our samples for one of the BOLD alleles. mtDNA haplotypes of *P. ignipectus* and *P. bicincta* also differ by <2% (Foottit et al., 2014).

### 3.3 The *w*Pig genome contains three divergent types of CI loci

We identified Type 1, 3, and 4 *cifs* in the *w*Pig genome (Martinez et al., 2020). This specific complement of *cifs* is not found in any other published *Wolbachia* genomes, but close relatives to each *w*Pig *cif* Type are. For instance, the *w*Pig Type 1 genes are 99% identical to those in the genome of the *Wolbachia* infecting the gall‐inducing wasp *Diplolepis spinosa* (Cynipidae), but less than 90% similar to any others (Martinez et al., 2020). The Type 3 *w*Pig genes are 99% identical to those in the genome of the *Wolbachia* infecting *D. spinosa*, the Staphylinid beetle *Diploeciton nevermanni*, and the water strider *Gerris buenoi*. The *w*Pig Type 4 genes are 99% identical to those in *Wolbachia* infecting *Nomada* bees (*w*NLeu, *w*NFla, and *w*NPa), but less than 95% identical to other Type 4 *cifs*. The *Wolbachia* infecting *D. spinosa* does not have Type 4 *cifs*, distinguishing it from *w*Pig. None of the *w*Pig *cifs* are truncated relative to copies with 99% identity. Additional sequencing is required to make more detailed *cif* comparisons.

### 3.4 Pervasive *w*Pig frequency variation

*w*Pig varied in frequency in several ways. First, frequency varied spatially among all samples (FET, *P* = 0.001) (Table 1), among sites in 2019 (FET, *P* < 0.0001), and 2020 (FET, *P* = 0.033). This variation occurred over a geographic radius of only 20 km in 2019 and 70 km in 2020 (Figure 3). Second, frequency varied across all samples between 2019 (*p* = 0.88 [0.82, 0.92]; *N* = 169) and 2020 (*p* = 0.95 [0.92, 0.97]; *N* = 317) (FET, *P* = 0.003). For the four sites we sampled in both years, frequencies were only significantly different between 2019 (*p* = 0.73 [0.56, 0.86]; *N* = 37) and 2020 (*p* = 1.0 [0.91, 1.0]; *N* = 40) in New Vineyard (FET, *P* < 0.001). Third, across all samples *w*Pig frequency was higher in females (*p* = 0.95 [0.93, 0.97]; *N* = 332) than males (*p* = 0.86 [0.80, 0.91]; *N* = 154) (FET, *P* = 0.001). However, this was driven mostly by a paucity of infected males in New Vineyard (males: *p* = 0.69 [0.50, 0.84], *N* = 32; females: *p* = 1.0 [0.92, 1.0], *N* = 45; FET, *P* < 0.0001), with no differences in *w*Pig frequency between males and females in other populations. *w*Pig frequency in males was relatively low in 2019 (*p* = 0.17 [0.02, 0.48]; *N* = 12), but fixed in 2020 (*p* = 1.0 [0.83, 1.0]; *N* = 20). We interpret these results as pervasive spatial, and rare temporal and sex‐specific, variation in *w*Pig frequency.

**TABLE 1:** *w*Pig infection frequencies in *Prosapia ignipectus* at each sampled site across both years

Site	GPS coordinates	*N*	Infected	*p* [Confidence Interval]
Carthage	44 36 44N, 70 28 10W	116	98	0.84 [0.77, 0.91]
New Portland	44 52 17N, 70 07 00W	72	68	0.94 [0.86, 0.98]
New Vineyard	44 45 14N, 70 08 01W	77	67	0.87 [0.77, 0.94]
Strong	44 47 08N, 70 13 42W	69	68	0.99 [0.92, 1.0]
Silver Lake	43 53 01N, 71 10 41W	20	19	0.95 [0.75, 1.0]
Dixfield	44 34 10N, 70 27 21W	41	41	1.0 [0.91, 1.0]
Weld	44 41 27N, 70 25 30W	33	32	0.97 [0.84, 1.0]
Wilton	44 37 58N, 70 18 10W	26	26	1.0 [0.87, 1.0]
Wonalancet	43 54 38N, 71 21 29W	32	31	0.97 [0.84, 1.0]

Note

Sample sizes (*N*), infection frequencies (*p*), and exact 95% binomial confidence intervals for each site.

## 4 DISCUSSION

Our results suggest that *w*Pig is a group‐B *Wolbachia* acquired after the initial divergence of *P. ignipectus* from *P. bicincta*. Analysis of *Wolbachia* and mtDNA haplotypes indicates that *w*Pig has no apparent effect on the *P. ignipectus* morphological contact zone in Maine. Across all samples, *w*Pig occurs at very high frequencies, consistent with our discovery of three divergent sets of CI loci in the *w*Pig genome. Finally, we document pervasive spatial, and rare temporal, *w*Pig frequency variation. We discuss this in more detail below.

### 4.1 *Wolbachia* acquisition in spittlebugs

In contrast to very high *w*Pig frequencies in *P. ignipectus*, we found no evidence of *Wolbachia* in our sample of 100 *P. bicincta*. A prior report of one infected *P. bicincta* sample indicates that *Wolbachia* could infect this species (Anderson et al., 2019). If so, it must be at very low frequencies, given our credible interval here (*p* = 0.0 [0.0, 0.04]; *N* = 100). Mathematical models predict that intense CI drives *Wolbachia* to high frequencies, balanced by imperfect maternal transmission (Hoffmann et al., 1990; Turelli & Hoffmann, 1995); conversely, *Wolbachia* that do not cause strong CI tend to occur at much lower frequencies (Cooper et al., 2017; Hague, Mavengere, et al., 2020; Hamm et al., 2014; Kriesner et al., 2016). While crossing to test for CI in the laboratory is not currently feasible in this system, the presence of three sets of CI loci in the *w*Pig genome, combined with its very high frequencies, suggests that *w*Pig causes intense CI.

How did *P. ignipectus* acquire *w*Pig? There are three possibilities: cladogenic transmission from its most recent common ancestor with its sister species, presumably *P. bicincta* or a close relative; by introgression from *P. bicincta* or another close relative; or by horizontal transmission (O’Neill et al., 1992). Given that we find no evidence for a high frequency *Wolbachia* in *P. bicincta*, cladogenic acquisition seems implausible, although we cannot fully rule it out. Without more extensive analysis of close relatives, we also cannot rule out introgression. However, opportunities for introgression with species other than *P. bicincta* have likely been limited. Other species of the genus *Prosapia* or family Cercopidae occur no further north than the US–Mexico border region, about 1,400 km from the nearest *P. ignipectus* populations and 3,000 km from the populations studied here.

Overall, the limited data are consistent with relatively recent noncladogenic transmission, a process that seems to be common among *Drosophila* species (Turelli et al., 2018). It may also be common among spittlebugs. This would be in stark contrast to obligate transovarial endosymbionts associated with amino acid nutrition in spittlebugs and other hemipterans (Koga et al., 2013). In addition to the thrip‐related *Wolbachia* found in *P. spumarius* in this study, Nakabachi et al. (2020) report that two spittlebug species, *Aphrophora quadrinotata* Say and *Philaenus maghresignus* Drosopoulos & Remane (both *Aphrophoridae*), harbor *Wolbachia* with 16S rRNA sequence that is identical to *Wolbachia* in two psyllid species, two whiteflies, an aphid, a planthopper, two leafhoppers, two grasshoppers, a mosquito, and a weevil. Likewise, Lis et al. (2015) report that *Wolbachia* they studied in *P. spumarius* is closely related to strains in vespids, drosophilids, whiteflies, chrysomelid beetles, and weevils based on five MLST loci. Kapantaidaki et al. (2021) also report *Wolbachia* infections at low levels in *P. spumarius*, as well as higher frequencies in *Neophilaenus campestris* (Fallén) (Aphrophoridae). Based on five MLST loci, their *N. campestris* strain is closely related to *Wolbachia* found in a leafhopper (Hemiptera) and cluster with *Wolbachia* from a planthopper, a scale insect and a psyllid (all Hemiptera), as well as two chrysomelid beetles, two butterflies, a parasitic wasp, and a mosquito. Koga et al. (2013, Table S2) report the presence of *Wolbachia* in the spittlebug *Cosmoscarta heros* (F.) (Cercopidae), in addition to *A. quadrinotata* and *P. maghresignus*.

In contrast, five specimens of *Poophilus costalis* (Walker) (Aphrophoridae) (Wiwatanaratanabutr, 2015), six specimens of *Philaenus tesselatus* Melichar (Lis et al., 2015), 37 specimens of *Philaenus signatus* Melichar (Kapantaidaki et al., 2021; Lis et al., 2015), and single specimens of *Philaenus arslani* Abdul‐Nour & Lahoud, *Philaenus loukasi* Drosopoulos & Asche, and *Philaenus tarifa* Remane & Drosopoulos (Lis et al., 2015) were not infected. Based on limited sequence data, the emerging pattern suggests that *Wolbachia* infection is widespread, but far from ubiquitous among spittlebugs, and that when it does occur, it often involves *Wolbachia* strains similar to those infecting distantly related insects. Whole *Wolbachia* and host genomic data are sorely needed to test our hypothesis that horizontal *Wolbachia* acquisition might be common in spittlebugs.

### 4.2 Little contribution of *w*Pig to the *P. ignipectus* morphological contact zone

We find no evidence for differentiation in *w*Pig or mtDNA haplotypes among *P. ignipectus* color forms. This includes the monomorphic black (New Vineyard) and lined (New Portland) populations that are separated by only 10 km in Maine, with no obvious barriers to dispersal or reproduction (Thompson & Carvalho, 2016). We also found no variation in *w*Pig or mtDNA haplotypes between black and lined individuals in the polymorphic Strong population. *w*Pig frequency also did not vary between color forms. These data indicate that *w*Pig is unlikely to significantly contribute to the maintenance of the *P. ignipectus* morphological contact zone.

How common are *Wolbachia* effects on host RI? Obligate *Wolbachia* infections in co‐occurring *D. paulistorum* semispecies contribute to assortative mating and generate hybrid inviability and male sterility (Miller et al., 2010). *Wolbachia* also contribute to reinforcement between *Wolbachia*‐infected *D. recens* and uninfected *D. subquinaria* (Jaenike et al., 2006; Shoemaker et al., 1999). In contrast, *Wolbachia* do not contribute to premating, gametic, or postzygotic RI among the three *D. yakuba*‐clade host species (Cooper et al., 2017). While the crossing schemes used in these *Drosophila* studies to dissect *Wolbachia* contributions to RI are not feasible in *P. ignipectus* and many other systems, our genetic data here lend support to our prior conjecture that *Wolbachia* contributions to RI observed in some *Drosophila* may be the exception rather than the rule (Cooper et al., 2017; Turelli et al., 2014).

### 4.3 Pervasive *w*Pig frequency variation

Mathematical models indicate that imperfect maternal transmission, *Wolbachia* fitness effects, and the severity of CI govern *Wolbachia* frequencies in host populations. *Wolbachia* that cause intense CI tend to occur at high and stable frequencies, balanced by imperfect maternal transmission (Barton & Turelli, 2011; Carrington et al., 2011; Hoffmann et al., 1990; Kriesner et al., 2013; Turelli & Hoffmann, 1991, 1995), while *Wolbachia* that cause weak or no CI tend to persist at intermediate, often variable frequencies (Cooper et al., 2017; Hague, Mavengere, et al., 2020; Hamm et al., 2014; Kriesner et al., 2016). Accumulating evidence for variable infection frequencies (Cooper et al., 2017; Hamm et al., 2014; Hughes, Allsopp, et al., 2011; Hughes, Ren, et al., 2011; Kriesner et al., 2016; Lis et al., 2015; Schuler et al., 2016), including our discovery here, highlights that infection frequencies are not static, even for high‐frequency variants.

With the exception of model systems like *w*Ri in *D. simulans*, few estimates of the key parameters required to approximate population frequency dynamics and equilibria of *Wolbachia* exist (Carrington et al., 2011; Turelli & Hoffmann, 1995). *w*Mel‐like *Wolbachia* frequencies in the *D. yakuba* clade vary through space and time in west Africa (Cooper et al., 2017), due in part to effects of cold temperatures on *w*Yak titer (Hague, Mavengere, et al., 2020). CI strength also varies in the *D. yakuba* clade, which may influence infection frequencies (Cooper et al., 2017; Hague, Caldwell, et al., 2020). *w*Mel frequencies vary with latitude in *D. melanogaster* populations, potentially due to *w*Mel fitness costs in the cold (Kriesner et al., 2016). Interestingly, hot temperatures reduce *w*Mel CI strength and transmission in transinfected *Aedes aegypti* used for biocontrol of human disease (Ross et al., 2017, 2020), suggesting that temperature may generally influence key parameters underlying *Wolbachia* infection frequencies.

What underlies variable *w*Pig frequencies in nature? High *w*Pig frequencies and the presence of three divergent sets of *cifs* suggest, but do not confirm, that *w*Pig causes strong CI. It seems plausible that some or all of these loci were horizontally acquired (Cooper et al., 2019), but additional sequence data are required to test this. We hypothesize that variable *w*Pig transmission rates contribute to the frequency variation we observe, potentially due to environmental effects on titer, as observed for *w*Yak (Hague, Mavengere, et al., 2020). Temporal variation in transmission was also observed for *w*Ri between two samples of *D. simulans* collected from Ivanhoe, California, in April and November of 1993 (Carrington et al., 2011; Turelli & Hoffmann, 1995), although the relative stability of *w*Ri frequencies in global *D. simulans* populations suggests that its transmission persists across a range of environmental conditions. Additional analyses of *Wolbachia* titer and transmission in the field, and across environmental contexts, are needed to better understand the causes of *Wolbachia* frequency variation. Comparing the titer and transmission of *Wolbachia* that occur at different frequencies in nature—for example, those that do and do not cause intense CI—would be particularly useful.

## CONFLICT OF INTEREST

We declare no conflicts of interests.

## AUTHOR CONTRIBUTIONS

**Timothy B. Wheeler:** Data curation (supporting); Investigation (lead); Validation (supporting); Visualization (lead); Writing‐original draft (supporting); Writing‐review & editing (supporting). **Vinton Thompson:** Conceptualization (equal); Data curation (supporting); Formal analysis (supporting); Investigation (equal); Methodology (equal); Project administration (supporting); Resources (equal); Visualization (supporting); Writing‐original draft (supporting); Writing‐review & editing (equal). **William R. Conner:** Data curation (supporting); Formal analysis (equal); Investigation (equal); Writing‐original draft (supporting); Writing‐review & editing (supporting). **Brandon Cooper:** Conceptualization (equal); Data curation (supporting); Formal analysis (equal); Funding acquisition (lead); Investigation (equal); Methodology (equal); Project administration (lead); Resources (equal); Supervision (lead); Validation (equal); Visualization (supporting); Writing‐original draft (lead); Writing‐review & editing (equal).

## Supporting information

Table S1

Table S2

## Data Availability

The *Wolbachia* infection frequency data, assemblies, and scripts are archived on DRYAD (https://doi.org/10.25338/b8ms7n). The base‐called Nanopore reads (SRR14328167), *Wolbachia* MLST sequences (MZ291996–MZ292028), and mitochondrial DNA sequences (MZ254780–MZ254784) are available a NCBI.
