# Supplementary material for: Wolbachia in the spittlebug Prosapia ignipectus: Variable infection frequencies, but no apparent effect on host reproductive isolation
Source: Ecol Evol. 2021 Jul 4;11(15):10054–65. doi: 10.1002/ece3.7782 (PMC8328426; doi:10.1002/ece3.7782)
Supplement: Supplementary file 1 — Table S1 [file ECE3-11-10054-s002.docx]

| **Appendix Table 1. Spittlebugs from *Schizachyrium scoparium,* Maine and New Hampshire, August 2019 and 2020** | | | | | | | | | | | | | | | | |  |  |  |  |  |  |  |  |
| --- | --- | --- | --- | --- | --- | --- | --- | --- | --- | --- | --- | --- | --- | --- | --- | --- | --- | --- | --- | --- | --- | --- | --- | --- |
|  |  |  |  |  |  |  |  |  |  |  |  |  |  |  |  |  |  |  |  |  |  |  |  |  |
|  |  |  |  |  |  | ***Prosapia ignipectus*** | | | | | | | | |  | **Other spittlebug species#** | | | | | | | |  |
|  |  |  |  |  |  |  |  |  |  |  |  |  |  |  |  | ***Philaenarcys*** | ***killa*** |  | ***Lepyronia*** | ***quadrangularis*** |  | ***Philaenus*** | ***spumarius*** |  |
|  |  |  |  |  |  | **Color form** | | | | | |  |  |  |  |  |  |  |  |  |  |  |  |  |
|  |  |  |  |  |  |  |  |  |  |  |  |  |  |  |  |  |  |  |  |  |  |  |  |  |
|  |  |  |  |  |  | **Lined*** | | | |  | **Black** |  | **Total** | **Total by** |  |  |  |  |  |  |  |  |  |  |
|  | **Coordinates** | |  |  |  | Fully | Partially | Pronotal | **Total with** | |  |  | **all color** | **locality** |  |  |  |  |  |  |  |  |  |  |
| **Locality** | North | West | **Date** | **Sex** |  | lined | lined | line only | **any lines** |  |  |  | **forms** | **both sexes** | |  |  |  |  |  |  |  |  |  |
|  |  |  |  |  |  |  |  |  |  |  |  |  |  |  |  |  |  |  |  | Quadrangularis | | | |  |
| **New Vineyard ME** | 44 45 14 | 70 08 01 | 23-Aug-19 | ♂ |  | 0 | 0 | 0 | 0 |  | **12** |  | **12** | **34** |  |  |  |  |  |  |  |  |  |  |
|  |  |  |  | ♀ |  | 0 | 0 | 0 | 0 |  | **22** |  | **22** |  |  |  |  |  |  |  |  |  |  |  |
|  |  |  | 20-Aug-20 | **♂** |  | 0 | 0 | 0 | 0 |  | **22** |  | **22** | **68** |  |  |  |  |  | **4** |  |  |  |  |
|  |  |  |  | ♀ |  | 0 | 0 | 0 | 0 |  | **46** |  | **46** |  |  |  |  |  |  | **2** |  |  | **5** |  |
| **New Portland ME** | 44 52 17 | 70 07 00 | 23-Aug-19 | ♂ |  | **39** | 0 | 0 | **39** |  | 0 |  | **39** | **114** |  |  |  |  |  |  |  |  |  |  |
|  |  |  |  | ♀ |  | **75** | 0 | 0 | **75** |  | 0 |  | **75** |  |  |  |  |  |  |  |  |  |  |  |
|  |  |  | 20-Aug-20 | ♂ |  | **20** | **1** | 0 | **21** |  | 0 |  | **21** | **41** |  |  |  |  |  |  |  |  |  |  |
|  |  |  |  | ♀ |  | **20** | 0 | 0 | **20** |  | 0 |  | **20** |  |  |  |  |  |  | **1** |  |  |  |  |
| **Strong ME** | 44 47 08 | 70 13 42 | 23-Aug-19 | ♂ |  | 0 | 0 | 0 | 0 |  | **6** |  | **6** | **65** |  |  |  |  |  |  |  |  |  |  |
|  |  |  |  | ♀ |  | **1** | **4** | **1** | **6** |  | **53** |  | **59** |  |  |  |  |  |  |  |  |  |  |  |
|  |  |  | 20-Aug-20 | ♂ |  | 0 | 0 | 0 | 0 |  | **14** |  | **14** | **32** |  |  |  |  |  | **5** |  |  |  |  |
|  |  |  |  | ♀ |  | 0 | **1** | 0 | **1** |  | **17** |  | **18** |  |  |  |  |  |  |  |  |  |  |  |
| **Carthage ME** | 44 36 44 | 70 28 10 | 23-Aug-19 | ♂ |  | **6** | **3** | **8** | **17** |  | **14** |  | **31** | **169** |  |  |  |  |  |  |  |  |  |  |
|  |  |  |  | ♀ |  | **13** | **38** | **21** | **67** |  | **66** |  | **138** |  |  |  |  |  |  |  |  |  |  |  |
|  |  |  | 20-Aug-20 | ♂ |  | **7** | 0 | **9** | **16** |  | **10** |  | **26** | **199** |  |  |  |  |  | **5** |  |  |  |  |
|  |  |  |  | ♀ |  | **8** | **34** | **55** | **97** |  | **76** |  | **173** |  |  |  |  |  |  | **1** |  |  |  |  |
| **Dixfield ME** | 44 34 10 | 70 27 21 | 20-Aug-20 | ♂ |  | **1** | **1** | **2** | **4** |  | **3** |  | **11** | **64** |  |  |  |  |  | **1** |  |  |  |  |
|  |  |  |  | ♀ |  | **2** | **2** | **7** | **11** |  | **31** |  | **53** |  |  |  |  |  |  | **3** |  |  |  |  |
| **Weld ME** | 44 41 27 | 70 25 30 | 20-Aug-20 | ♂ |  | 0 | 0 | 0 | 0 |  | **11** |  | **11** | **33** |  |  |  |  |  |  |  |  |  |  |
|  |  |  |  | ♀ |  | 0 | 0 | 0 | 0 |  | **22** |  | **22** |  |  |  |  |  |  |  |  |  |  |  |
| **Wilton ME** | 44 37 58 | 70 18 10 | 20-Aug-20 | ♂ |  | 0 | 0 | 0 | 0 |  | **2** |  | **2** | **27** |  |  |  |  |  |  |  |  |  |  |
|  |  |  |  | ♀ |  | 0 | 0 | 0 | 0 |  | **25** |  | **25** |  |  |  |  |  |  |  |  |  | **1** |  |
| **Wonalancet NH** | 43 54 38 | 71 21 29 | 17-Aug-20 | ♂ |  | 0 | 0 | 0 | 0 |  | **10** |  | **10** | **32** |  |  |  |  |  | **1** |  |  |  |  |
|  |  |  |  | ♀ |  | 0 | 0 | 0 | 0 |  | **22** |  | **22** |  |  |  |  |  |  | **1** |  |  |  |  |
| **Silver Lake NH** | 43 53 01 | 71 10 41 | 9-Aug-20 | ♂ |  | 0 | 0 | 0 | 0 |  | **13** |  | **13** | **20** |  | **2** |  |  |  |  |  |  |  |  |
|  |  |  |  | ♀ |  | 0 | 0 | 0 | 0 |  | **7** |  | **7** |  |  | **2** |  |  |  |  |  |  |  |  |
| **West Ossipee NH** | 43 50 15 | 71 11 20 | 9-Aug-20 | ♂ |  | 0 | 0 | 0 | 0 |  | **4** |  | **4** | **4** |  | **33** |  |  |  | **1** |  |  |  |  |
|  |  |  |  | ♀ |  | 0 | 0 | 0 | 0 |  | 0 |  | 0 |  |  | **48** |  |  |  |  |  |  |  |  |
|  |  |  |  |  |  |  |  |  |  |  |  |  |  |  |  |  |  |  |  |  |  |  |  |  |
| *Note 1. Fully lined individuals had a single transverse orange line on the pronotum and two complete lines across the wings. Partially lined individuals were missing one wing line or had one or two interrupted or incomplete wing lines. Pronotum line only individuals lacked wing lines but possessed the pronotal line in full or in partially obscured form. | | | | | | | | | | | | | | | | | | | | | | | |  |
|  |  |  |  |  |  |  |  |  |  |  |  |  |  |  |  |  |  |  |  |  |  |  |  |  |
| #Note 2. Other spittlebug species are recorded only for the 2020 collections, which included all specimens in this category tested for the presence of *Wolbachia*. | | | | | | | | | | | | | | | | | | |  |  |  |  |  |  |
