# Supplementary material for: Wolbachia in the spittlebug Prosapia ignipectus: Variable infection frequencies, but no apparent effect on host reproductive isolation
Source: Ecol Evol. 2021 Jul 4;11(15):10054–65. doi: 10.1002/ece3.7782 (PMC8328426; doi:10.1002/ece3.7782)
Supplement: Supplementary file 2 — Table S2 [file ECE3-11-10054-s001.docx]

| Appendix Table 2. Primers used in this study. | | |
| --- | --- | --- |
| **Primers** | | |
| **Primer Name** | **Sequence (5’-- 3’)** | **Reference** |
| **Mitochondrial CO1** | | |
| LepF | 5′-ATTCAACCAATCATAAAGATATTGG-3′ | Hebert et al. (2004a) |
| LepR | 5′-TAAACTTCTGGATGTCCAAAAAATCA-3′ | Hebert et al. (2004a) |
| **MLST** | | |
| coxA_F1 | 5'-TTGGRGCRATYAACTTTATAG-3' | Baldo et al. 2006 |
| coxA_R1 | 5'-CTAAAGACTTTKACRCCAGT-3' | Baldo et al. 2006 |
| gatB_F1 | 5'-GAKTTAAAYCGYGCAGGBGTT-3' | Baldo et al. 2006 |
| gatB_R1 | 5'-TGGYAAYTCRGGYAAAGATGA-3' | Baldo et al. 2006 |
| hcpA_F1 | 5'-GAAATARCAGTTGCTGCAAA-3' | Baldo et al. 2006 |
| hcpA_R1 | 5'-GAAAGTYRAGCAAGYTCTG-3' | Baldo et al. 2006 |
| fbpA_F1 | 5'-GCTGCTCCRCTTGGYWTGAT-3' | Baldo et al. 2006 |
| fbpA_R1 | 5'-CCRCCAGARAAAAYYACTATTC-3' | Baldo et al. 2006 |
| fbpA_F3 | 5′‐GTTAACCCTGATGCYYAYGAYCC‐3′ | Baldo et al. 2006 |
| fbpA_R3 | 5′‐TCTACTTCCTTYGAYTCDCCRCC‐3′ | Baldo et al. 2006 |
| wsp_F1 | 5'-GTCCAATARSTGATGARGAAAC-3' | Baldo et al. 2006 |
| wsp_R1 | 5'-CYGCACCAAYAGYRCTRTAAA-3' | Baldo et al. 2006 |
| ftsZunif | 5'-GGYAARGGTGCRGCAGAAGA-3' | Lo et al. 2002 |
| ftsZunir | 5'-ATCRATRCCAGTTGCAAG-3' | Lo et al. 2002 |
| **Wolbachia infection screen** | | |
| wsp_pcr_F | 5′‐TGGTCCAATAAGTGATGAAGAAAC‐3′ | Braig et al. 1998 |
| wsp_pcr_R | 5′‐AAAAATTAAACGCTACTCCA‐3′ | Braig et al. 1998 |
| 28s_pcr_F | 5′‐TACCGTGAGGGAAAGTTGAAA‐3′ | Choudhury & Werren, 2006 |
| 28s_pcr_R | 5′‐AGACTCCTTGGTCCGTGTTT‐3′ | Choudhury & Werren, 2006 |
